# Influence of urban green open space on residents’ physical activity in China

**DOI:** 10.1186/s12889-019-7416-7

**Published:** 2019-08-13

**Authors:** Han Wang, Xiaoling Dai, Jinglan Wu, Xingyi Wu, Xin Nie

**Affiliations:** 1 School of Public Administration, Guangxi University, Nanning, Guangxi China; 2 Department of City and Regional Planning, University of North Carolina at Chapel Hill, Chapel Hill, USA; 3 China Construction Fourth Bureau Construction Development Co., Ltd., Xiamen, Fujian China

**Keywords:** Green open space, Physical activity, Social ecology theory, Order probit

## Abstract

**Background:**

Urban green open space is a valuable resource for physical activities of urban inhabitants and has the potential to reduce chronic illness and improve health. Research on the relationships between green open space and physical activity is incomplete and limited in China. Thus, the study examines how the urban green open space contributes to physical activity.

**Methods:**

A questionnaire was designed based on the social ecology theory to investigate the physical activity of 513 residents in urban green open space. We use the time and frequency of residents exercising in urban green space to measure physical activity, and use the factor analysis to synthesize a large number of original factors (i.e., infrastructure, safety, accessibility, landscape quality, and space environment) into relatively few composite indicators. Based on the collected data of the cross-sectional population, the Order Probit regression model was constructed to analyze how urban green open space affects the residents’ physical activity from the perspective of social ecology.

**Results:**

① in community factors: accessibility is significantly positive correlation with residents’ physical activity, and there is no significant correlation between safety and physical activity; ②in natural factors: space environment and landscape quality are not significantly correlated with residents’ physical activity; ③ in built environmental factors: infrastructures, the area of green space, the size of open space, and entertainment facilities are significantly correlated to residents’ activity. Basketball courts, volleyball courts, swimming pools, and sports equipment will promote physical activity; ④ apart from the attributes of green open space, other factors are significantly correlated to physical activity in the green open space, e.g. having a companion.

**Conclusions:**

Urban green open space plays an important role in promoting physical activity especially among the women and the old, and improving the attributes (such as accessibility, infrastructures, the area of green space, the size of open space and entertainment facilities) of the urban green open space and trying to set up group sports proper to play with companion (like “square dancing” and “Tai Chi”) can promote Chinese residents’ physical activity so as to improve public health. The results are significant to facilitate environment health.

## Background

With increasing urbanization, the obesity rate, overweight rate, and chronic disease mortality rate have increased consistently, thus forming a major global public health issue. The most recent US research report showed that about 2.2 billion people worldwide are overweight, accounting for one-third of the world’s total population. Furthermore, about 712 million people (10% of the global population) are obese [1]. The number of obese people is 106 million in the US and 93.8 million in China. Researchers pointed out that the global goal set by the World Health Organization (not to exceed the 2010 level of obesity by 2025) is almost impossible to reach [2]. In addition, chronic diseases account for 60% of all deaths, 80% of which occur in low-and middle-income countries (including China), where chronic disease deaths account for three-quarters of all global chronic disease deaths [3]. With the urbanization and industrialization, China has become the world’s second largest economy; however, Chinese residents’ sedentary behavior (such as going out with the car, watching TV, and using computers) has constantly increased and their physical activity (such as walking and physical exercise) has decreased. According to Chinese Nutrition and Chronic Diseases Report (CNCDR), the overweight rate of Chinese adults is 30.1%, and the obesity rate is 11.9%, indicating increases of 7.3 and 4.8% since 2002, respectively [4]. In addition, in 2012, the death rate for chronic diseases in China was 533/100,000, accounting for 86.6% of the total number of deaths [4].

One-third of adults are physically inactive in the world [5], and a number of studies have shown that physical inactivity not only causes overweight [6], obesity [7, 8], and chronic diseases [9], but also increases the risk of cancer and premature death [10]. Furthermore, a study by the University of Cambridge in the United Kingdom reported that maintaining moderate physical activity (PA) was key to reduce the risk of premature death. The World Health Organization (WHO) identified physical inactivity as the fourth leading risk factor for global mortality [11]. Therefore, PA contributes to human health. PA has been reported to reduce obesity [9], cardiovascular diseases [12], and mental health problems [13]. Furthermore, PA was also reported to promote health [14], reduce stress [15], and increase mental well-being [16].

To improve people’s PA level, it is very important to know learn which factors are associated with PA. For the study of the influencing factors of individual behavior, social ecology theory has constructed a very good theoretical framework. The theory was first proposed by Bronfenbrenner, and was further developed by McLeroy et al. [17]. The social ecology theory distinguishes the influence of five fields on individual behavior: personal factors, interpersonal factors, social factors, natural factors, and environmental factors. Most studies investigated PA from a single dimension and focused on the analysis of the impact of personal factors [18] or environmental factors [19] on PA. With the development and the social ecology theory, it was gradually realized that many other factors will also affect individual PA, which can be more in line with the habits of individual PA through policy or planning [18].

Urban green open spaces (such as parks) are a common space for PA, which can be used by a wide range of people to improve their health [20, 21]. However, studies on the association between parks and physical activity have used a mixture of self-reports and objective measurements, which may lead to mixed results. Urban green open space has been reported to help promote physical activity [6] and reduce a variety of chronic illness [22], and a positive relationship among them has been found [6, 23–25]. Li et al. [23] reported a positive relationship between built environment factors (density of places of employment, household density, green and open spaces for recreation, and number of street intersections) and walking activity. Liu et al. [24] reported a positive correlation of urban parks with public PA as well as positive mental health benefits. Akpinar and Cankurt [25] analyzed the associations between characteristics of urban green spaces and the frequency of PA in the city of Aydın, Turkey, and reported that for the general population, a short distance to urban green spaces, many trees, the availability of exercise equipment, and picnic areas were positively associated with the frequency of PA, while barbecues and fire places were negatively associated with the duration of PA.

Other researchers reported that large and attractive parks could promote walking for health benefits [20, 26]. Moreover, safety [27], better access to green spaces, and park facilities may be associated with higher use and in turn increased participation in physical activity [6, 28]. However, a number of studies found no relationship between urban green open space and PA [18, 28], and several studies found there was no relationship between distance, park size, and PA [28, 29]. Coombes et al. [6] measured the availability of green space within the neighborhoods of participants, and concluded that the availability of green space was not associated with PA. Potwarka et al. [30] found no relationship between the proximity to parks and overweight. In general, the evidence for an association between green space and PA is mixed. Most studies found positive associations, while few either reported no or negative associations. Although the relationship between these is controversial, many cities have incorporated the idea of increasing urban green space to encourage PA [24].

In summary, urban green open space is a valuable resource for the improvement of human health by encouraging more participation in PA to reduce morbidity. In China in particular, people are increasingly interested in the positive impact of green space on PA and other health benefits. Liu et al. [24] studied the relationship between Beijing urban parks, PA, and mental health, and found a positive correlation. However, this study only selected a single type of green space in urban parks, and only used personal factors (gender, age, and occupation) and environmental factors (accessibility). Environmental factors also did not include information about park characteristics, such as park size or infrastructure. Moreover, the impact of natural factors (landscape quality), interpersonal factors (company and family), and social factors (security) on PA were not included; therefore, further research on this topic in China is needed. In addition, the obtained research conclusions of urban planning and PA in western countries are not necessarily applicable to Chinese cities. As the largest developing country and the second largest economy of the world, China applies its own particular urban planning system, which includes a considerable amount of urban green space, as well as unique national conditions and customs.

Since the Chinese economy currently enters a stable and favorable trend, under the policy of “adhering to the people-centered development thinking, vigorously developing a healthy cause, and being a healthy nation”, the Central Government works toward improving the quality of life of the residents by building urban green open spaces and emphasizing the vital role of maintaining a healthy 76lifestyle. Given the Chinese preference for outdoor group sports, China uses urban green open space as the main driving force to enhance the residents’ level of PA. According to statistics, the green area of urban built-up areas in China has reached 1,971,000 ha in 2016, which constitutes an increase of 20.6%; furthermore, the per capita green area of the open space has reached 13.5 m^2^, constituting an increase of 9.8% [31]. Therefore, the urban green open space for residents’ fitness and leisure has already been formed in China.

The relationship between urban green space and PA is not only of academic interest, but also provides important significance for improving public health and rational urban land planning. This provides both reference and guidance for the planning of urban green open spaces, including the size of open space, landscape, and infrastructure. However, the empirical basis of these policy recommendations is often weak [32]. Consequently, more research is needed to better quantify and describe the optimal plan of green open space, especially for the promotion of PA to improve resident health. Early studies focused on Western developed countries; however, people from different socio-economic strata and cultural backgrounds use green space differently, and their PA behavior is affected by different factors. Therefore, developing countries also need corresponding empirical research [33]. Related academic research however, remains limited research in China.

At present, China has entered a new era that emphasizes green open spaces and has been facing a contradiction between the people’s growing need for a better life and the inadequate development and imbalance of their physical fitness. Therefore, the rational use of green open space for PA has become an important means for improving the physical quality of Chinese residents. The objective of this study is to analyze the influence of all types of urban green open spaces on residents’ physical activity using social ecology theory.

The following research questions were explored: (1) Among social and ecology factors, which factors are significantly associated with PA? (2) How is PA influenced by social and ecology factors? (3) What is the unique of PA pattern and urban green open space use among Chinese?

## Methods

### Research theory

The social ecology theory originated from the ecosystem theory, originally proposed by Bronfenbrenner, who identified the natural environment as the main source of human development, and the developing individual as in the middle of several environmental systems that span from a direct environment (similar to a family) to an indirect environment (similar to a broad culture) [17, 34]. In 1988, McLeroy et al. expanded on this idea, indicating that individuals, families, interpersonal relationships, and community were factors that affected the health behavior of individuals [17, 34]. In 1992, Stokols proposed a social ecology model that introduced both nature and society as vital elements that influence individual behavior [17, 34]. In 2005, Zimring et al. introduced the built environment into the social ecology model as one of the factors that affect personal behavior; this established the social ecology theory [34]. The social ecology theory suggests individual behavior to be affected by social ecological factors; it has multiple dimensions including individuals, families, society, nature, and the environment. The social ecology theory has overcome the limitation of analyzing individual behavior from a single perspective and instead emphasizes that individual behavior is affected by numerous factors. Firstly, at the individual level, individual self-awareness, age level, salary level, and educational level all impact individual behavior. Secondly, at the interpersonal level, the family either encourages or discourages the individual and friends either provide support or not. Whether any interaction between the individual and society exists will play a catalytic or inhibitory role on the individual behavior. Thirdly, at the community level, the community’s security, culture, and community activities will impact the individual behavior. Fourthly, at the natural level, the venues, air quality, and the types of vegetation will all attract individuals to varying degrees and will lead to different individual behaviors. Finally, at the built environmental level, the characteristics of activity sites, such as infrastructure, facilities, and the area of activity sites are important factors to promote individual behavior.

Since then, the social ecology theory has been widely applied for the improvement of heart health, to prevent gluten-related diseases as well as HIV/AIDS, to intervene in juvenile delinquency, to promote community health management, and for the analysis of their effects on the PA of students or older adults [17, 34–39]. Therefore, to explore the impact of social ecological factors on individual behavior in the environment, this study conducted an empirical investigation of the factors that affect the residents’ PA behavior in the urban green open space. Following the central idea of the social ecology theory that individual behavior is affected by social ecological factors, this study divided the influencing factors of urban green open space on residents’ PA into five major categories of social ecological factors: individual factors, interpersonal factors, community factors, natural factors, and built environmental factors.

### Study area

**Fig. 1:**
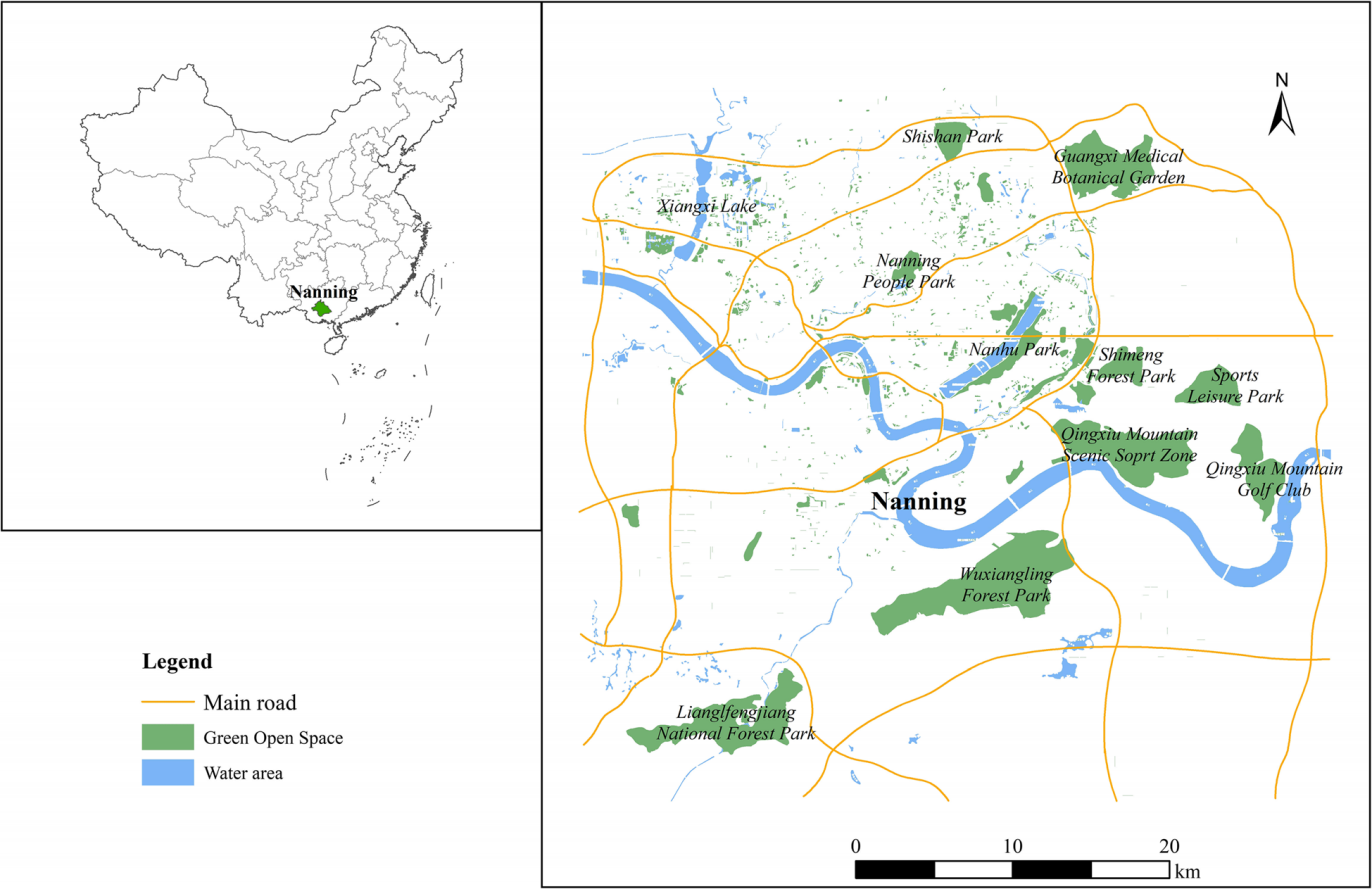
Study areas. (The author drew this map by ArcGIS10.2 software)

Nanning is the capital of Guangxi Province, China, and is located within 22°13′ and 23°32′N latitude and 107°45′ and 108°51′E longitude (Fig. 1). It is an emerging city that integrates economy, politics, culture, society, and ecology. Nanning has a humid subtropical monsoon climate with an abundance of sunshine. Since 2013, it has been ranked among the top ten cities of China’s air quality ranking and has been named the “Green City”. According to People’s Daily reported in 2015, more than 90% of cities in China were in sub-health, while Nanning has been healthy since 2012 [40]. It attracts a large floating population. By the end of 2016, its green area reached 106,294.2 ha and the green area of the built-up area reached 10,704.57 ha. The green park area reached 12.01 m^2^/person and the quality of the Green City achieved a substantial increase [41]. In January 2016, Nanning officially became the first National Ecological Garden City in China [42]. From 1994 to 2017, Nanning’s urban green open spaces increased from 13 to 38 [41], has providing its residents with a valuable resource for physical activity. However, like other cities in China, the residents lack the awareness and rational utilization of these resources, and they also lack adequate exercise and sufficient health literacy. A survey of the state of health of residents in 2015 found that more than 80% of the residents of Nanning City were generally healthy, while 30% of residents suffered from chronic diseases. Therefore, Nanning was selected as a representative study area to analyze the impact of green open space on residents’ PA.

### Data sources of the questionnaire

According to China’s green space classification standard [43], the types of green open spaces include urban green parks, square spaces, and waterfront spaces. These also differ in functions, scales, and service ranges in each sub-category within each category. Therefore, to improve the diversity of samples and the achieved representation, stratified sampling of all green open spaces in proportion to their size was selected. Finally, a total of 20 green open spaces were selected, including comprehensive parks, special parks, residential parks, children’s parks, country parks, wetland parks, wildlife parks, amusement parks, outdoor playgrounds, neighborhood recreation parks, sightseeing agricultural park, and the city square. For the purposes of this study, data was specifically gathered. Apart from the Nanning city land bureau institute of green spot figure data, the classic method of questionnaire assessment was used. The questions of the questionnaire were designed based on the social ecology theory (Table 1). To make the questionnaire representative, residents who were using the urban green open space for physical activity were invited to participate in the study via face to face interview from June to October in 2016. A total of 574 questionnaires were sent out and 539 questionnaires were received. 513 questionnaires were valid and the resulting effective rate was 89.84%.

**Table 1:** Main content of the questionnaire

Variable	Social ecology theory	Evaluation elements	Description	Variable classification
Dependent variables		Physical Activity time	Time range: below 30 min to 4 h and above	Sequencing the dependent variable
		Physical Activity frequency	Frequency range: every year, month, week, day	Sequencing the dependent variable
Independent variables	Individual factors	Age	Adults	Continuous variable
		Gender	Male and female	Dummy variable (1 = male, 0 = female)
		Wage	2000 RMB to 20,000 RMB or more	Continuous variable
	Interpersonal factors	Companion	Family, classmates and friends, colleagues, a single person, team, relatives, others	Dummy variable (1 = family, 2 = classmates or friends, 3 = colleagues, 4 = single, 5 = team, 6 = relatives, 7 = others)
	Community factors	Accessibility	Public transportation is convenient, distance to settlements, time and money spent, entrances and exits set	Factor variable
		Safety	Barrier-free design, related logo, night lighting, security patrols	Factor variable
	Natural factors	Space environment	Air noise, cleanliness, vegetation coverage, environmental compatibility	Factor variable
		Landscape quality	Rich vegetation, rich landscape changes, good natural scenery	Factor variable
	Built environment factors	Entertainment facilities	Race track, swimming pool, chess room, stone road, area for square dance or kung fu, Tai Chi, roller skating field, sports equipment and venue	Dummy variable (1 = yes, 0 = none)
		The area of green open space	Range: 0–1250 m^2^	Continuous variable
		Size of open space	Range: 1.88–4666.67 ha	Continuous variable
		Infrastructure	Toilets, shelters, tables, and chairs, facilities, and convenience stores	Dummy variable

The questionnaire contained two parts, including PA time and PA frequency and social ecology dimensions about the impact of urban green open space on PA. Among these questions, five indicators (i.e., safety, accessibility, landscape quality, space environment, and infrastructure) were described by a number of indicators of factors. The Likert Five Point Scale was used and options of those factors were provided, where “strongly disagree, disagree, uncertain, agree, and strongly agree” were associated with scores of “1, 2, 3, 4, 5”, respectively. Then, respondents were asked to rate each factor and the obtained numbers were used to reflect the impact of green open space on the residents’ PA. All major variables are listed in Table 1.

### Questionnaire

#### Dependent variables

According to the purpose of this study, the PA time and the PA frequency were selected as dependent variables to represent the level of PA.

PA Time: the time of residents’ PA in a green open space. This includes less than 30 min, from 30 min to 1 h, from 1 h to 2 h, from 2 h to 4 h, and 4 h and above.

PA Frequency: the times of residents’ PA in a green open space. This gradually increases, ranging from once per year, once per month, once or more times per week, to once or more times per day.

#### Independent variables

According to the social ecology theory, the factors that affect the residents’ PA in the urban green open space can be divided into several dimensions, such as community, nature, and the built environment. For this study, infrastructure, landscape quality, space environment, entertainment facilities, the area of green space, and the size of green open space were selected as independent variables. Specific descriptions are as follows:

- ① Community dimension:

Security: the surrounding law, order, threat, and patrolling of the green open space.

Accessibility: the degree of difficulty residents face to reach the green open space.

- ② Natural dimension:

Landscape quality: the natural landscape and air quality of green open space.

Space environment: the environmental cleanliness and air noise of the green open space.

- ③ Built environment dimension:

Infrastructure: basic bathrooms, tables, and chairs, convenience stores, and other facilities in the green open space.

Entertainment facilities: stadium, swimming pool, square dance, and runway of the urban green open space.

Size of the green open space: the size of the green open space in the study area.

Area of the green space: the per capita green area of potential users in a green open space.

### Control variables

Since other factors may indirectly influence residents’ physical activity in the urban open space, several control variables were considered. These were classified into individual factors and interpersonal factors, such as, gender, wage, and companion.

## Methods

### Factor analysis

The purpose of factor analysis was to synthesize a large number of original variables into relatively few composite indicators, i.e., factors. Since several of the characteristics of the green open space in this study are composed of categories of comprehensive indicators (i.e., infrastructure, safety, accessibility, landscape quality, and space environment) those indicators are described by a number of small indicators. Each of these is scored via the Likert Five Point Scale method, and factor analysis is used for simple calculation.

### Order probit model

For the dependent variables, the PA time gradually increased within 30 min and the physical activity frequency also increased from once per year to once per month, until it reaches multiple times per day. Both are multi-classification discrete values, that are ordered accordingly, but not continuously. Therefore, they belong to sequencing dependent variables. The Order Probit model was used to analyze these. It has the following general form:

1 $$ {\mathrm{Y}}_i=\beta {X}_i+{\varepsilon}_i $$

Where Y*i* represents a potential variable, *Xi* represents a set of independent variables, *β* represents the parameter to be estimated, and *ε*_*i*_ represents a random disturbance item.

### Gaussian floating catchment area method

The area of green space refers to the service value of green area with a spatial distance threshold of either 2.5 km or 5 km. The value is calculated via the first step of a Gaussian two-step moving search. First, the spatial distance threshold *d*_0_ was determined for each green open space. Then, this forms a spatial scope. Under normal circumstances, the walking speed of people is 5 km/h, the speed of rail traffic is 10 km/h, and people’s travel time generally does not exceed 30 min. Therefore, the spatial distance threshold can be assumed as 2.5 km or 5 km. Secondly, for each street *k* population fall within the spatial scope, and the Gaussian equation with weights and add these weighted populations were used. A number of potential users in the green open space *j* was obtained. Finally, the size of green open space was divided by the number of potential users, which yielded the ratio of supply and demand. Finally, multiplying the ratio of supply and demand by the population of the street *k*, obtained the maximum green space in the space *R*_*kj*_ [44].

2 $$ {R}_j=\frac{S_j}{\sum_{k\in \left\{{d}_{kj\le d\;0}\right\}}G\left({d}_{kj},{d}_0\right){P}^k} $$

Where *R*_*j*_ represents the per capita green area of potential users in the *j* space of the green open space, and *P*^*k*^ represents the population of the street *k* in the spatial extent (*d*_*kj*_ ≤ *d*_0_) of the green open space *j*. *d*_*kj*_ represents the spatial distance from the center of street *k* to the center of street *j*. *S*_*j*_ represents the capacity of the green space. This study focuses on the area of each green open space. *G*(*d*_*kj*_, *d*_0_) represents a Gaussian equation that considers the problem of space friction. It is calculated with the following formula:

3 $$ G\left({d}_{kj},{d}_0\right)=\left\{\frac{e^{-\left(\frac{1}{2}\right)\times {\left(\frac{d_{kj}}{d_0}\right)}^2}-{e}^{-\left(\frac{1}{2}\right)}}{\begin{array}{l}1-{e}^{-\left(\frac{1}{2}\right)}\\ {}0,{d}_{kj}>{d}_0\end{array}}\right.,{d}_{kj}\le {d}_0 $$

## Results

### Data reliability

Cronbach’s reliability analysis was conducted using the STATA13.0 software. It shows that the reliability of the total scale (all of the variables) reached 0.8621, indicating that the factors of the questionnaire is credible.

### Data validity

Then, Bartlett’s spherical test and KMO value analysis were performed. The results obtained a *P* value of 0.000 (*P* < 0.001), indicating that the model passes the Bartlett’s test of sphericity. The KMO value was 0.879, which is above 0.60. Thus, the sample data is suitable for factor analysis.

### Order probit model for green open spaces on physical activity

#### The model results of green open space on residents’ PA time

Model 1 of Tab. 2 is based on the residents’ “PA time” as the dependent variable Order Probit regression model. The results of model 1 indicate that green open space whose infrastructure, area of green space, size of green open space, and facilities significantly impact residents’ PA time. The model results for different variables are shown as follows:

**Table 2:** Order Probit model of physical activity estimation results

	Model 1: Physical activity time			Model 2: Physical activity frequency		
Variable	Regression coefficients	Standard deviation	Exponent sign	Regression coefficients	Standard deviation	Exponent sign
Age	0.108	0.0672	1.11	0.117^a^	0.0680	1.12
Gender	−0.370^c^	0.104	0.69	0.0446	0.102	1.05
Wage	0.066^a^	0.040	1.06	0.0201	0.0446	1.02
Companion _ classmates or friend	0.933	0.144	2.54	0.176	0.136	1.19
Companion _ colleagues	0.570^c^	0.178	1.77	0.104	0.184	1.11
Companion _ single	−0.135	0.205	0.87	0.413^b^	0.180	1.51
Companions _ team	1.024^c^	0.272	2.78	0.141	0.898	1.15
Companion _ relatives	−0.063	0.315	0.94	−0.233	0.284	0.79
Companions _ others	0.410	0.261	1.51	0.451^a^	0.255	1.57
Accessibility	0.0017	0.076	1.002	0.292^c^	0.0738	1.34
Safety	−0.137	0.086	0.87	−0.0223	0.0710	0.97
Space environment	−0.006	0.0820	0.99	−0.0458	0.0763	0.96
Landscape quality	−0.029	0.073	0.97	−0.0561	0.0715	0.95
Infrastructure	0.220^c^	0.0794	1.25	−0.107	0.0745	0.90
Area of green space	−0.0003998^a^	0.00022	0.999	0.000225	0.000251	1.01
Size of open space	0.000107^a^	0.000057	1.01	−0.000178^c^	6.34e-05	0.99
Is there - skating field	−0.329^b^	0.151	0.72	−0.365^b^	0.175	0.69
Is there - basketball court	0.610^c^	0.151	1.84	−0.00304	0.182	0.99
Is there - volleyball court	1.268^c^	0.259	3.55	0.864^c^	0.248	2.37
Is there - stone road	0.06	0.208	1.06	−0.169	0.190	0.84
Is there - swimming pool	0.466^b^	0.200	1.59	−0.00170	0.219	0.99
Is there - tennis court	−0.346	0.240	0.71	−0.151	0.249	0.86
Is there - sports equipment	0.170	0.135	1.19	0.475^c^	0.134	1.61
Is there - table tennis court	−1.293^c^	0.280	0.27	−0.558^b^	0.252	0.58

Please note: ^a^, ^b^, and ^c^, indicate significance levels of 10, 5, and 1%, respectively. (Data were collected from questionnaires)

(1) The community factors of urban green open space affect residents’ PA time: Accessibility and safety have no significant effect on residents’ PA time; however, the regression coefficient of accessibility indicates that accessibility plays a positive role in promoting physical activity.

(2) The natural factors of urban green open space affect residents’ PA time: The impact of landscape quality and space environment on residents’ PA time is insignificant.

(3) The built environment factors of urban green open space affect residents’ PA time: ① Infrastructure and PA time are significantly and positively correlated at the 1% level. ② A significant negative correlation was found between the area of green space and the PA time at the 5% level. ③ A significant positive correlation was found between the size of green open space and the PA time at the 10% level. ④ For the design of entertainment facilities, all surveyed green open spaces in cities are designed with runway, “square dance”, and “Tai Chi”; green open spaces with basketball courts, volleyball courts, and swimming pools increase the resident’s PA time. Green open spaces with a table tennis court are significant negatively correlated with PA time.

(4) The control variables in urban green open space affect residents’ PA time: Of the individual factors, gender is significantly correlated with PA time at the 10% level. Wage is significantly positively correlated with the PA time at the 1% level. With regard to interpersonal factors, a significant positive correlation was found between PA time and colleagues or a team at the 1% level.

#### The model results of green open space on residents’ physical activity frequency

Model 2 is based on residents “physical activity frequency” as the dependent variable in the Order Probit regression model. The model results of different variables are shown as follows:

(1) The community factors of urban green open space affect residents’ PA frequency: The model results indicate that there is no significant correlation between safety and the physical activity frequency. Accessibility is positively correlated with the PA frequency at the 1% level.

(2) The natural factors of urban green open space affect residents’ PA frequency: Space environment and landscape quality are not significantly related to PA frequency.

(3) The built environmental factors of urban green open space affect residents’ PA frequency: ① The infrastructure is not significantly correlated to the PA frequency. ② The effect of the per capita green space on the PA frequency is not significant. ③ A significant negative correlation was found between the size of green open space and the residents’ PA frequency the 1% level. ④ In entertainment facilities, both the air volleyball court and sports equipment are significantly positive correlated with PA frequency at the 1% level. However, whether there is a skating field and whether there is a table tennis field have a significant negative correlation with the PA frequency at the 5% level.

(4) The control variables in urban green open space affect residents’ PA frequency: For individual factors, age and residents’ physical activity frequency are significantly and positively correlated at the 10% level. For interpersonal factors, the physical activity frequency is significantly correlated with being alone or accompanied by others at the 5 and 10% levels, respectively.

## Discussion

### Community factors of urban green open space on physical activity

Accessibility refers to the ease of transport, the distance from the place of residence to green open space, and the time spent in transit, which is an important factor of PA in the green open space [6, 12, 45]. An easier accessibility of the green open space leads to higher willingness of residents to go there for PA. The obtained results verified this. When the accessibility increases every unit, the probability of high frequency of residents’ PA increases by 34%, indicating easier access of residents for the green open space leads participation in PA. This is mainly related to the improvement of traffic conditions in recent years in Nanning. With the opening of Nanning metro lines 1 and 2, the convenience of residents to go out has been strongly improved, and the time spent on the road was also constantly shortened; thus, the activity frequency increased.

The safety of the urban green open space ensures PA [46] and can be viewed as an important factor for why people use urban green space [45]. Safety inhibits PA time to a certain extent, which shows that the law and order in Nanning is good and the actions of pickpockets and related crimes are under control; therefore, the safety of people in the green open space is protected. Adding more security patrols would create social unrest among the residents who stay there, creating the mentality that the longer they stay, the greater the threat for their own security.

### The natural factors of urban green open space on physical activity

Studies showed that natural elements such as grass, trees, or flowers, cleanliness, maintenance, and aesthetics of the urban green open space were associated with PA [26, 45, 47]. The results of this study differed from the above viewpoints due to the wide variety of green plants, the beautiful natural scenery, clean environment, and fresh air of Nanning green open space. The natural landscapes are generally in a relatively stable environment. Therefore, these factors had no significant effect on the residents’ PA.

### The built environment factors of urban green open space on physical activity

According to the results of Table 2:① Infrastructure is significantly correlated to the PA time and not to the PA frequency. Urban green open space with more features, such as latrines, rest kiosks, shelters, convenience stores, and toilets, satisfy people’s basic physiological needs, and thus enable them to spend a longer time here and make it more likely to use the green open space for PA [28, 45]. ② For the area of green space, the results showed that if all other variables remain unchanged, the area of green space increased by one hectare and the probability that residents choose a lower activity frequency would increase by 99%. In previous studies, the total area of urban green open space was significantly associated with PA [28], which was consistent with the findings of the current study. This is because living indoors for a long time, people look forward to embrace nature. In return, green plants can absorb the CO_2_ exhaled by people and convert this to energy and O_2_ through photosynthesis. In addition, it is known that some green plants can also absorb CO and SO_2_ from the air as well as other toxic gases. Trees and other green plants block dust via filtration and absorption. The most important factor is that green plants affect the metabolism by producing a large number of negative ions directly in the central nervous system, inducing feelings of happiness in people [24]. In short, green plants can make people feel comfortable and energetic, and thus, change the functional structure to resist a variety of infectious diseases. Despite the many benefits brought by green plants, those green plants take up a considerable area of green open space. More plants reduce the area for available PA, which restricts the range of residents’ PA. ③ The size of green open space has a significant positive correlation with PA time, which is consistent with the results of previous studies [26]. However, it has a significant negative correlation with PA frequency. This is because a larger space increases the per capita activity area; therefore, residents will stay longer [48]. After finishing basic sports such as walking, if residents play chess or walk on gravel or jump square dance and other activities, the residence time would increase, which leads to a decrease of the PA frequency compared to the “limited free time” of residents. ④ The design of the entertainment facilities in the urban green space has a significant association with PA [18]. The present study showed that all surveyed green open spaces in cities are designed with runway, “square dance”, and “Tai Chi”, and those group sports are very popular with Chinese residents. Apart from these activities, the green open space with basketball courts, volleyball courts, and swimming pools increase the resident’s PA time. In addition to basic facilities, if there are more facilities, residents will spend more time trying those and consequently increase the time for PA. However, since Nanning is in the subtropical zone, the swimming pool is more popular. Basketball is one of the most influential ball games in the world and is the most favorite sport for most boys. The green open space with a basketball court increases the probability of residents choosing longer PA time. Volleyball has become a favorite sport for all Chinese since it is aimed at people of all works of life and includes men and women of all ages. Equipping green open spaces with a volleyball court can increase the probability of residents choosing longer PA time by 255% and increase the PA frequency by 137%. The green open space with table tennis court is significantly negatively correlated with PA, which is because the regional habits of Nanning are different.

The probability that residents choose higher PA frequency in the green open space with sports equipment is 61% higher than for those without sports equipment, which is similar to the findings of previous studies [18, 26]. However, the presence of a skating field is significantly negatively correlated with PA frequency. Residents have a 69% higher probability of selecting lower PA frequency in the green open space without a skating field than with a skating field. Furthermore, residents have a 58% higher probability of selecting lower PA frequency in a green open space without table tennis than with table tennis. This is mainly because the green open space provides entertainment facilities for PA. If the facilities have not been used for a short time, people will choose to try them one or more times separately. The main activity areas for roller skating are frequented by both children and young adults. As the floor area of the roller-skating site is large, once the participants fall, it is easy to hurt themselves and the people nearby. Therefore, to a certain extent, their PA frequency is reduced.

### The control variables in urban green open space on physical activity

With regard to individual factors, the probability of women choosing longer PA time is higher than that of men, indicating that women have more time for leisure activities than men [49]. This is inseparable from the family status of women. Most women bear children, cook at home, and conduct other housework; therefore, they spend more time for PA in neighborhood urban green space [28], while men have less leisure time, because they are the main monetary providers in China. Residents with high salary can increase the probability of choosing longer PA time [50]. When all other variables remain unchanged, the probability for residents to choose a high PA frequency in green open space will increase by 12% with each additional year of age. This finding is different from the results of Evenson et al. [49] who reported that older adults were infrequently observed in the parks of Western cities [49], but our findings show the characteristic of Asian cities [45]. This is mainly because as residents age, their awareness of a pursuit of quality life will increase and they also focus more on enjoying life. In particular the retired seniors have more free time to enjoy their life. Furthermore, these are the most important patrons of the green open space for square dance, playing ball, running, and playing chess. Therefore, this shows that the time spent doing PA increases with age.

With regard to interpersonal factors, residents who choose colleagues or another team to engage in physical activity have a 77 and 178% increased probabilities of choosing longer PA time, indicating that team and colleagues’ support are the predominant source of social support for individuals to participate in PA [51]. This further reflects the existence of the “group consciousness” among Chinese residents. These findings match those of Schetke et al. [45] who have reported that visitors of green areas enjoy being together with friends and families for group activities in two Asian cities. However, whether PA is conducted alone or with a partner will increase the PA frequency. This is because individuals have more flexibility in their personal time; therefore, they will go to the open space to exercise if this can be done flexibly. Moreover, being with a partner will increase the motivation compared to PA alone.

The main contribution of our study is that it fills a gap in the existing literature that uses a single dimension to study the influencing factors of a park on PA. This study analyzed the influencing factors of all types of urban green space on PA from multiple dimensions based on the social ecology theory. A further contribution is that this study is the first to systematically analyze the influencing factors (i.e., community factors, natural factors, built environment factors, individual factors, and interpersonal factors) on Chinese residents’ PA. A third contribution is that this study designed the questionnaire based on the social ecology theory, and both the data as well as the investigated city are representative.

However, this study has several limitations. The PA was self-reported, therefore, the PA time and PA frequency may be overestimated compared to the real PA level. It was not possible to determine whether the reported frequency and time of PA were a valid measure of actual use. However, this practice is similar to that of Jackson [52] who has shown acceptable levels of validity for single-response items for PA estimation. A further limitation is that the study is cross sectional in nature, therefore, it is difficult to determine if the observed relationships are causal.

## Conclusion and recommendations

The ecological civilization construction aims to promote environments and physical activity as a part of daily life, but research has so far provided mixed results between urban green open space and physical activity. Besides, previous research has mainly been conducted on western developed countries, those findings cannot simply be transposed to the eastern developing context where PA form and urban green open space are quite different. Our study was both innovative in combining the social ecology theory with the questionnaire and was policy-relevant. Based on our results among the respondents in Nanning, suggestions for the planners of urban green open space in China can be made.

For community factors, no significant positive correlation was found between accessibility and the PA time, but a significant positive correlation was found to PA frequency. Consequently, improving the accessibility of urban green open spaces can encourage more residents to participate in PA to increase their use of green space and enhance the health of residents. Moreover, maintaining the safety of the urban green open space and shortening the distance from residence to the green open space can ensure the security of residents and reduce the time spent on the road, which can further promote PA.

For natural factors, the space environment and landscape quality have a positive effect on residents’ PA. Therefore, the urban green open space should be planted with a variety of vegetation types to green the open space, to freshen the air in the green open space, and to regulate residents in the green open space.

For built environmental factors, infrastructures, the area of green space, the size of the open space, whether a basketball court exists or a volleyball court, and a swimming pool are all significantly positively correlated with residents’ PA time, while the existence of a table tennis court is significantly negatively correlated with PA time. A significant positive correlation was found between the presence of a volleyball court or entertainment facilities and residents’ PA frequency. The size of green open space, a rolling field and a table tennis field have a significant negative correlation with PA frequency. This is because Chinese residents have a strong preference for group sports, which leads to all green open spaces being specially designed as “square dancing” and “Tai Chi” squares. Consequently, expanding the per capita area of urban green space, adding swimming pools and adding ethnic characteristics and culture to sports activities can promote residents’ PA.

Apart from the attributes of the green open space, there are also several other factors that influence residents’ PA in the urban green open space: individual and interpersonal factors. In individual factors, age and PA time as well as PA frequency are significantly and positively correlated; and gender is significantly correlated to PA time. The majority of the PA groups in China are elderly, and women spend more time with PA than men. In interpersonal factors, a strong sense of community exists in China, and choosing colleagues, a team, or others for group sports can promote PA. Therefore, to improve the health of the entire population, the urban green open space should be designed with a number of additional group sports that are suitable for both men and young people in particular.

## Data Availability

The data we analyzed in the manuscript came from our questionnaire survey. And the data used and analyzed during the current study are available from the corresponding author on reasonable request.

## Abbreviations

PA: physical activity

## References

[1] Sina tech: The latest US data: 33% of the global population is overweight, 10% obese. http://www.techweb.com.cn/data/2017-06-15/2535258.shtml. Accessed 15 June 2017.

[2] Obesity overall population and the world rankings, are you fat? Please use BIM table. https://baijiahao.baidu.com/s?id=1572505194270341&wfr=spider&for=pc. Accessed 10 Jul 2017.

[3] Interpretation: Current Status of Chronic Diseases in China. http://www.360doc.com/content/16/0113/18/2522440_527654355.shtml. Accessed 13 Jan 2016.

[4] Chinese Nutrition and Chronic Diseases Report (2015). http://www.sohu.com/a/113182293_381036. Accessed 5 July 2015.

[5] Hallal PC, Andersen LB, Bull FC, Guthold R, Haskell W, Ekelund U, lancet physical activity series working group (2012). Global physical activity levels: surveillance progress, pitfalls, and prospects. Lancet.

[6] Coombes E, Jones AP, Hillsdon M (2010). The relationship of physical activity and overweight to objectively measured green space accessibility and use. Soc Sci Med.

[7] Shaw KA, Gennat HC, O'Rourke P, Del Mar C. Exercise for overweight or obesity. Cochrane Database Syst Rev. 2006;4. 10.1002/14651858.CD003817.pub3 PMC9017288 17054187

[8] Sallis JF, Floyd MF, Rodríguez DA, Saelens BE (2012). Role of built environments in physical activity, obesity, and cardiovascular disease. Circulation.

[9] Nocon M, Hiemann T, Müller-Riemenschneider F, Thalau F, Roll S, Willich SN (2008). Association of physical activity with all-cause and cardiovascular mortality: a systematic review and meta-analysis. Eur J Cardiovasc Prev Rehabil.

[10] Sobering Statistics on Physical Inactivity in the U.S. https://www.sciencedaily.com/releases/2015/08/150826093015.htm. (2015).

[11] WHO: Global recommendations on physical activity for health (2010). 26180873

[12] Tamosiunas A, Grazuleviciene R, Luksiene D, Dedele A, Reklaitiene R, Baceviciene M, et al. Accessibility and use of urban green spaces, and cardiovascular health: findings from a Kaunas cohort study, Environmental Health. 2014;13(1):20. 10.1186/1476-069X-13-20 PMC4000006 24645935

[13] Mackay GJ, Neill JT (2010). The effect of “green exercise” on state anxiety and the role of exercise duration, intensity, and greenness: a quasi-experimental study. Psychol Sport Exerc.

[14] Koohsari MJ, Mavoa S, Villanueva K, Sugiyama T, Badland H, Kaczynski AT, Giles-Corti B (2015). Public open space, physical activity, urban design and public health: Concepts, methods and research agenda. Health Place.

[15] Thompson CW, Roe J, Aspinall P, Mitchell R, Clow A (2012). Miller, D.:more green space is linked to less stress in deprived communities: evidence from salivary cortisol patterns. Landsc Urban Plan.

[16] Wood L, Hooper P, Foster S, Bull F (2017). Public green spaces and positive mental health – investigating the relationship between access, quantity and types of parks and mental wellbeing. Health Place..

[17] Huang H, Zhang JK (2016). A systematic review of related research on Adolescents' physical activity in Western countries based on the perspective of social-ecology. China Sports Sci.

[18] Schipperijn J, Bentsen P, Troelsen J, Toftager M, Stigsdotter UK (2013). Associations between physical activity and characteristics of urban green space. Urban For Urban Green.

[19] Duncan M, Mummery K (2005). Psychosocial and environmental factors associated with physical activity among city dwellers in regional Queensland. Prev Med.

[20] Cohen DA, McKenzie TL, Sehgal A, Williamson S, Golinelli D, Lurie N (2007). Contribution of public parks to physical activity. Am J Public Health.

[21] De Jong K, Albin M, Skärbäck E, Grahn P, Björk J (2012). Perceived green qualities were associated with neighborhood satisfaction, physical activity, and general health: results from a cross-sectional study in suburban and rural scania, southern Sweden. Health Place.

[22] World Bank: Climate change, disaster risk, and the urban poor (2011).

[23] Li F, Fisher KJ, Brownson RC, Bosworth M (2005). Multilevel modelling of built environment characteristics related to neighbourhood walking activity in older adults. J Epidemiol Community Health.

[24] Liu H, Li F, Li J, Zhang Y (2017). The relationships between urban parks, residents' physical activity, and mental health benefits: a case study from Beijing, China. J Environ Manag.

[25] Akpinar A, Cankurt M (2017). How are characteristics of urban green space related to levels of physical activity: examining the links. Indoor Built Environ.

[26] Sugiyama T, Francis J, Middleton NJ, Owen N, Giles-Corti B (2010). Associations between recreational walking and attractiveness, size, and proximity of neighborhood open spaces. Am J Public Health.

[27] Amorim TC, Azevedo MR, Hallal PC (2010). Physical activity levels according to physical and social environmental factors in a sample of adults living in South Brazil. J Phys Act Health.

[28] Kaczynski AT, Potwarka LR, Saelens BE (2008). Association of park size, distance, and features with physical activity in neighborhood parks. Am J Public Health.

[29] Hillsdon M, Panter J, Foster C, Jones A (2006). The relationship between access and quality of urban green space with population physical activity. Public Health.

[30] Potwarka LR, Kaczynski AT, AL F (2008). Places to play: association of park space and facilities with healthy weight status among children. J Commun Health.

[31] We have walked through the extraordinary five years. http://news.cctv.com/2017/08/11/ARTIYFeZUxtMWMuVIPSlBtAV170811.shtml. Accessed 17 August 2017.

[32] Veal A (2013). Open space planning standards in Australia: in search of origins. Aust Plan.

[33] Kabisch N, Qureshi S, Haase D (2015). Human–environment interactions in urban green spaces—a systematic review of contemporary issues and prospects for future research. Environ Impact Assess Rev.

[34] Zimring C, Joseph A, Nicoll GL, Tsepas S (2005). Influences of building design and site design on physical activity: research and intervention opportunities. Am J Prev Med.

[35] Doran K, Resnick B, Kim N, Lynn D, McCormick T (2017). Applying the social ecological model and theory of self-efficacy in the worksite heart health improvement project-PLUS. Res Theory Nurs Pract.

[36] Whittemore R, Melkus GDE, Grey M (2004). Applying the social ecological theory to type 2 diabetes prevention and management. J Community Health Nurs.

[37] Hong JS, Voisin DR, Crosby S (2015). A review of STI/HIV interventions for delinquent and detained juveniles: an application of the social–ecological framework. J Child Fam Stud.

[38] Langille JLD, Rodgers WM (2010). Exploring the influence of a social ecological model on school-based physical activity. Health Educ Behav.

[39] Kang S, Wang W, Cole ST. Using Social Ecological Factors to Measure the Social Benefits of Leisure Activity on Senior Adults’ Quality of Life (QOL): A Validation in 2013. Natl Senior Games. 2016; (In Chinese).

[40] Zhang SP, Xu B, Meng JX (2015). The evaluation on urban ecosystem health and its obstacle degree:a case study on Nanning city. J Anhui Univ (Natural Sciences).

[41] Guangxi statistical yearbook. Bureau of statistics of the Guangxi Zhuang Autonomous Region, China http://www.gxtj.gov.cn/tjsj/tjnj/2017/indexch.htm. Accessed 14 Sep 2017.

[42] Nanning is the first national ecological garden city to be the only provincial capital city. Nanning news 30 January 2016.

[43] Shen, D. X., Xiong G. P.: About Urban Green Open Space. Urban Plann Bull. 1996; (6), 7–11+6–64.(In Chinese).

[44] Wang Y, Xu CL, Gao R, Wang Q (2014). Evaluation of green space accessibility of Shenyang using Gaussian based 2-step floating catchment area method. Prog Geogr.

[45] Schetke S, Qureshi S, Lautenbach S, Kabisch N (2016). What determines the use of urban green spaces in highly urbanized areas?–examples from two fast growing Asian cities. Urban For Urban Green.

[46] Carver A, Timperio A, Crawford D (2008). Playing it safe: the influence of neighbourhood safety on children's physical activity—a review. Health Place.

[47] McCormack GR, Rock M, Toohey AM, Hignell D (2010). Characteristics of urban parks associated with park use and physical activity: a review of qualitative research. Health Place..

[48] Paquet C, Orschulok TP, Coffee NT, Howard NJ, Hugo G, Taylor AW, Daniel M (2013). Are accessibility and characteristics of public open spaces associated with a better cardiometabolic health?. Landsc Urban Plan.

[49] Evenson KR, Jones SA, Holliday KM, Cohen DA, McKenzie TL (2016). Park characteristics, use, and physical activity: a review of studies using SOPARC (system for observing play and recreation in communities). Prev Med.

[50] Dai, J., Chen, H., Li, J., Zhang, J.K., Li, N.: Factors to influence the adolescents’ Sport & Health Behaviors from the perspective of social ecology. J Shanghai Univ Sport 2017; 41: 35–41.(In Chinese).

[51] Chen, Na., Wang, C.Q.: Research on health behavior of elderly in newly-arising disadvantage urban communities based on PRECEDE-PROCEED model. Acta Universitatis Medicinalis Nanjing (Social Sciences). 2016; 16, 442–445.(In Chinese).

[52] Jackson AW, Morrow JR, Bowles HR, FitzGerald SJ, Blair SN (2007). Construct validity evidence for single-response items to estimate physical activity levels in large sample studies. Res Q Exerc Sport.

